# MRI radiomics enhances radiologists’ ability for characterizing intestinal fibrosis in patients with Crohn’s disease

**DOI:** 10.1186/s13244-024-01740-6

**Published:** 2024-06-28

**Authors:** Mengchen Zhang, Yinghou Zeng, Zhuang-nian Fang, Yang-di Wang, Ruo-nan Zhang, Ziyin Ye, Qing-hua Cao, Ren Mao, Canhui Sun, Zhi-hui Chen, Bingsheng Huang, Xue-hua Li

**Affiliations:** 1 Department of Radiology, The First Affiliated Hospital, Sun Yat-Sen University, Guangzhou, People’s Republic of China; 2 Medical AI Lab, School of Biomedical Engineering, Medical School, Shenzhen University, Shenzhen, People’s Republic of China; 3 Department of Pathology, The First Affiliated Hospital, Sun Yat-Sen University, Guangzhou, People’s Republic of China; 4 Department of Gastroenterology, The First Affiliated Hospital, Sun Yat-Sen University, Guangzhou, People’s Republic of China; 5 Gastrointestinal Surgery, The First Affiliated Hospital, Sun Yat-Sen University, Guangzhou, People’s Republic of China

**Keywords:** Crohn’s disease, Fibrosis, Radiomics, MR enterography

## Abstract

**Objectives:**

We aimed to develop MRI-based radiomic models (RMs) to improve the diagnostic accuracy of radiologists in characterizing intestinal fibrosis in patients with Crohn’s disease (CD).

**Methods:**

This retrospective study included patients with refractory CD who underwent MR before surgery from November 2013 to September 2021. Resected bowel segments were histologically classified as none-mild or moderate-severe fibrosis. RMs based on different MR sequence combinations (RM1: T2WI and enhanced-T1WI; RM2: T2WI, enhanced-T1WI, diffusion-weighted imaging [DWI], and apparent diffusion coefficient [ADC]); RM3: T2WI, enhanced-T1WI, DWI, ADC, and magnetization transfer MRI [MTI]), were developed and validated in an independent test cohort. The RMs’ diagnostic performance was compared to that of visual interpretation using identical sequences and a clinical model.

**Results:**

The final population included 123 patients (81 men, 42 women; mean age: 30.26 ± 7.98 years; training cohort, *n* = 93; test cohort, *n* = 30). The area under the receiver operating characteristic curve (AUC) of RM1, RM2, and RM3 was 0.86 (*p* = 0.001), 0.88 (*p* = 0.001), and 0.93 (*p* = 0.02), respectively. The decision curve analysis confirmed a progressive improvement in the diagnostic performance of three RMs with the addition of more specific sequences. All RMs performance surpassed the visual interpretation based on the same MR sequences (visual model 1, AUC = 0.65, *p* = 0.56; visual model 2, AUC = 0.63, *p* = 0.04; visual model 3, AUC = 0.77, *p* = 0.002), as well as the clinical model composed of C-reactive protein and erythrocyte sedimentation rate (AUC = 0.60, *p* = 0.13).

**Conclusions:**

The RMs, utilizing various combinations of conventional, DWI and MTI sequences, significantly enhance radiologists’ ability to accurately characterize intestinal fibrosis in patients with CD.

**Critical relevance statement:**

The utilization of MRI-based RMs significantly enhances the diagnostic accuracy of radiologists in characterizing intestinal fibrosis.

**Key Points:**

- MRI-based RMs can characterize CD intestinal fibrosis using conventional, diffusion, and MTI sequences.
- The RMs achieved AUCs of 0.86–0.93 for assessing fibrosis grade.
- MRI-radiomics outperformed visual interpretation for grading CD intestinal fibrosis.

**Graphical Abstract:**

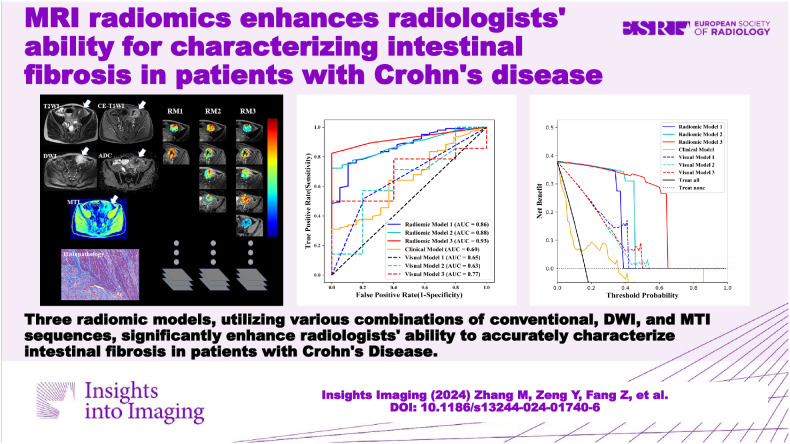

## Introduction

Fibrostenosis is a severe complication of Crohn’s disease (CD) that significantly impacts patients’ quality of life. Currently, intestinal fibrosis is recognized as a dynamic and reversible condition rather than a static and irreversible entity [1–4]. None-mild fibrosis may be reversed by medical treatment [4]. Unfortunately, there are no effective medical interventions for moderate-severe intestinal fibrosis, which necessitates endoscopic or surgical treatment [1–4]. Accurate detection and grading of intestinal fibrosis severity is crucial for selecting appropriate treatments and may facilitate the development of novel antifibrotic therapies.

As transmural fibrosis cannot be detected through endoscopic visualization or partial-thickness biopsy, cross-sectional imaging serves as a non-invasive means of evaluating fibrostenosis [5]. However, conventional imaging remains inadequate or yields inconsistent results in the evaluation of intestinal fibrosis [6–8]. While magnetization transfer magnetic resonance imaging (MTI) has demonstrated potential in accurately evaluating intestinal fibrosis [7, 9], concerns persist regarding interobserver variability and operator subjectivity. The full benefits of MTI for diagnosing fibrosis cannot be realized by relying solely on a single parameter and conventional delineations with limited areas and regions of interest. To date, there is no consensus on the standard for evaluating intestinal fibrosis in CD.

In recent years, radiomics analysis has increasingly been utilized in medical images to enhance the assessment of CD [10–12]. Radiomics extracts large amounts of high-dimensional features from images to uncover disease characteristics that may not be discernible through visual inspection or single-parameter analysis [13, 14]. In a previous study [15], a novel computed tomography enterography (CTE) radiomic model (RM) was developed and validated allowing for accurate characterization of intestinal fibrosis in CD. However, because CTE has the potential risk of radiation, magnetic resonance enterography (MRE) is the preferred follow-up examination for patients with CD. Moreover, multi-sequence MRE performs better than CTE in the assessment of intestinal fibrosis [5–7]. Tabari et al reported that texture analysis of contrast-enhanced imaging can detect bowel fibrosis in CD [10]. However, this study only included small sample size (*n* = 25). A recent study has explored the assessment of intestinal fibrosis in a mouse model through textural analysis of T2WI [11]. To the best of our knowledge, no existing study has investigated the efficacy of multi-sequence MRE-based radiomic analysis for evaluating CD intestinal fibrosis in humans.

This study aims to develop and verify MRE-based RMs for the diagnosis of intestinal fibrosis, with the objective of improving radiologists’ diagnostic accuracy and efficiency in characterizing this condition.

## Materials and methods

### Patients

From November 2013 to September 2021, we included 128 consecutive eligible patients with CD from our institution. This retrospective study was approved by the institutional ethics review board, which waived the requirement for obtaining informed consent.

The inclusion criteria were as follows: (a) patients diagnosed with CD based on standard clinical, imaging, endoscopic, and histological investigations [16]; (b) availability of preoperative MRE within three months of surgery for bowel strictures, fistula, or abscess; (c) availability of a histopathologic bowel segment corresponding to a matching affected intestine on MRE. The exclusion criteria were as follows: (a) inadequate imaging quality resulting from moving artefacts; (b) targeted bowel segment presenting with other bowel diseases, or located at an anastomosis; and (c) unidentifiable intestinal contour on MRE due to severe pericentric effusion, intestinal adhesion, or intestinal peristalsis.

The final enroled patients were randomly allocated into training and test cohorts in an approximate ratio of 3:1 for developing and validating the RMs, respectively.

### Surgical histopathology evaluation of intestinal fibrosis

We adopted a region-to-region positioning approach [8] to achieve accurate location matching of bowel segments between MRE and surgical specimens and histological sections (Supplementary Materials). One bowel segment was obtained for each patient.

Histological sections were collectively assessed by two pathologists (Z.Y. and Q.C., with 11–12 years of experience in bowel pathology and no access to clinical and radiological information) to reach a consensus. Scores 0–2 were considered none-mild, and scores 3–4 were considered moderate to severe (Supplementary materials). The ratio of none-mild to moderate-severe histopathology fibrotic samples in both the training and test cohorts remained consistent, ensuring that the diagnostic performance of the models was unbiased and robust.

### Development and validation of MRE-based RMs

#### MRE protocol

MRE was conducted on two 3-T MR systems (Magnetom Trio or Prisma; Siemens Healthineers) equipped with 12-(Trio) or 18-channel (Prisma) phased-array body coils, including T2WI, T1WI, diffusion-weighted magnetic resonance imaging (DWI), apparent diffusion coefficient (ADC), and MTI (details of the MRE protocol shown in Supplementary Materials; Table 1).

**Table 1:** MRI sequences and parameters

Parameter	T2WI HASTE (MR1/MR2)	DWI (MR1/MR2)	VIBE (MR1/MR2)	VIBE (MR1/MR2)	MT GRE (MR1/MR2)
Orientation	2D axial/2D axial	2D axial/2D axial	3D coronal/3D coronal	3D axial/3D axial	2D axial/2D axial
Acquisition matrix	320 × 224/320 × 243	132 × 132/134 × 134	320 × 240/320 × 256	320 × 240/320 × 165	256 × 205/256 × 205
Flip angle (°)	160/160	–	13/10.5	13/9	30/30
Slice thickness (mm)	4/4	4/4	2/2	2/3	4/4
Echo time (ms)	86/81	71/57	1.34/1.35	1.37/1.30	2.81/2.81
Repetition time (ms)	800/1000	5300/6800	4.37/4.28	4.37/3.31	230/232
Number of slices	28/45	28/45	80/88	88/72	10/10
Respiratory control	Breath-hold	Free-breathing	Breath-hold	Breath-hold	Breath-hold
Acquisition time (s)	24/45	159/130	28/22	24/18	30/26

*HASTE* half-Fourier acquisition single-shot turbo spin echo, *DWI* diffusion-weighted magnetic resonance imaging, *VIBE* volumetric interpolated breath-hold examination, *2D* two-dimensional, *3D* three-dimensional, *MT GRE* magnetization transfer gradient recalled echo, *MR1* magnetom trio, *MR2* magnetom prisma, *T2WI* T2-weighted imaging

#### Bowel segmentation

**Fig. 1:**
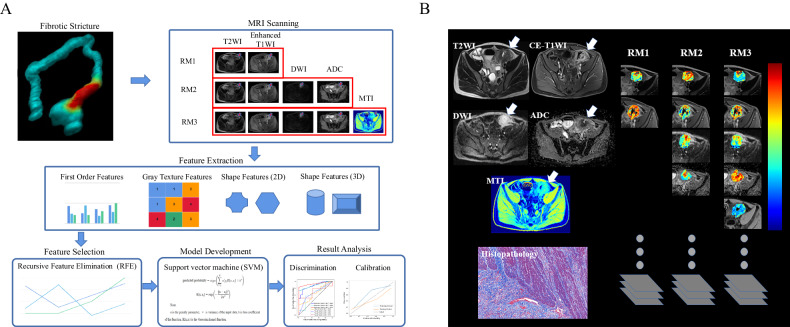
**A** The radiomics analysis workflow: RM1, RM2, and RM3 established by different sequence combinations aid in the evaluation of CD fibrosis. **B** A 41-year-old man with CD with moderate-severe fibrotic stricture in descending colon. Radiomics features extracted from different sequence combinations of T2WI, contrast-enhanced T1WI, DWI, ADC, and MTI are used to construct RM1–RM3. To generate feature maps, the features are calculated for each voxel in the segmented VOIs with a kernel radius of 3, and assigned to the center pixel. ADC, apparent diffusion coefficient; CD, Crohn’s disease; DWI, diffusion-weighted imaging; MTI, magnetization transfer MRI; RM, radiomic model

We have chosen T2WI, contrast-enhanced T1WI in the venous phase, DWI, ADC, and MTI for radiomics analysis. These standardized scanning sequences are recommended by the Society of Abdominal Radiology, the European Crohn’s and Colitis Organization, and the European Society of Gastrointestinal Radiology for patients with CD [6, 17]. Three RMs established by different sequence combinations (RM1: T2WI and enhanced-T1WI; RM2: T2WI, enhanced-T1WI, DWI, and ADC; RM3: T2WI, enhanced-T1WI, DWI, ADC, and MTI) were applicable to institutions equipped with varying MRI device conditions across different regions and levels of development (Fig. 1). Three-dimensional volumes of interest (VOIs) were manually segmented on the bowel lesions in each MRE sequence in the training and test cohorts by a gastrointestinal radiologist (M.Z.) with seven years of experience, using MITK (version 2018.04; https://www.mitk.org/). The VOIs for the entire region were delineated along the lesion contour on each sequence’s images, excluding the intestinal lumen. These completed VOIs served as masks to select voxels within the lesion.

#### Features extraction

We extracted the MRE radiomic features using the pyradiomics toolkit (https://pyradiomics.readthedocs.io/en/latest/installation.html). For reproducibility tests, two gastrointestinal radiologists (M.Z. with seven years of experience and Z.F. with three years) annotated additional VOIs on bowel segments in the training and test cohorts. The radiologist (M.Z.) conducted an intra-observer reproducibility test with a three-month interval between readings.

#### Features selection and RM development

Radiomic features were selected by recursive feature elimination (RFE) [18], with optimized parameters, including the weight of the feature importance determined by 10-fold cross-validation.

Based on the selected radiomic features, a binary classification RM using the support vector machines (SVM) [19] classifier was built to distinguish between none-mild and moderate-severe intestinal fibrosis (supplementary materials). The workflow of the radiomics analysis is shown in Fig. 1.

#### RM validation

RMs were validated using a completely independent test cohort. The diagnostic performance was evaluated using the area under the receiver operating characteristic curve (AUC) and calibrated using the Hosmer–Lemeshow goodness-of-fit test. Integrated discrimination improvement (IDI) and net reclassification improvement (NRI) were used to test for performance improvement among different RMs.

The impact of factors including the severity of inflammation (none-mild or moderate-severe inflammation), lesion locations (small bowel or colon), different MRI scanners (Magnetom Trio or Prisma; Siemens Healthineers), and bowel lesions with and without penetrating diseases on RM diagnostic performance was investigated.

### Development and validation of visual interpretation

**Fig. 2:**
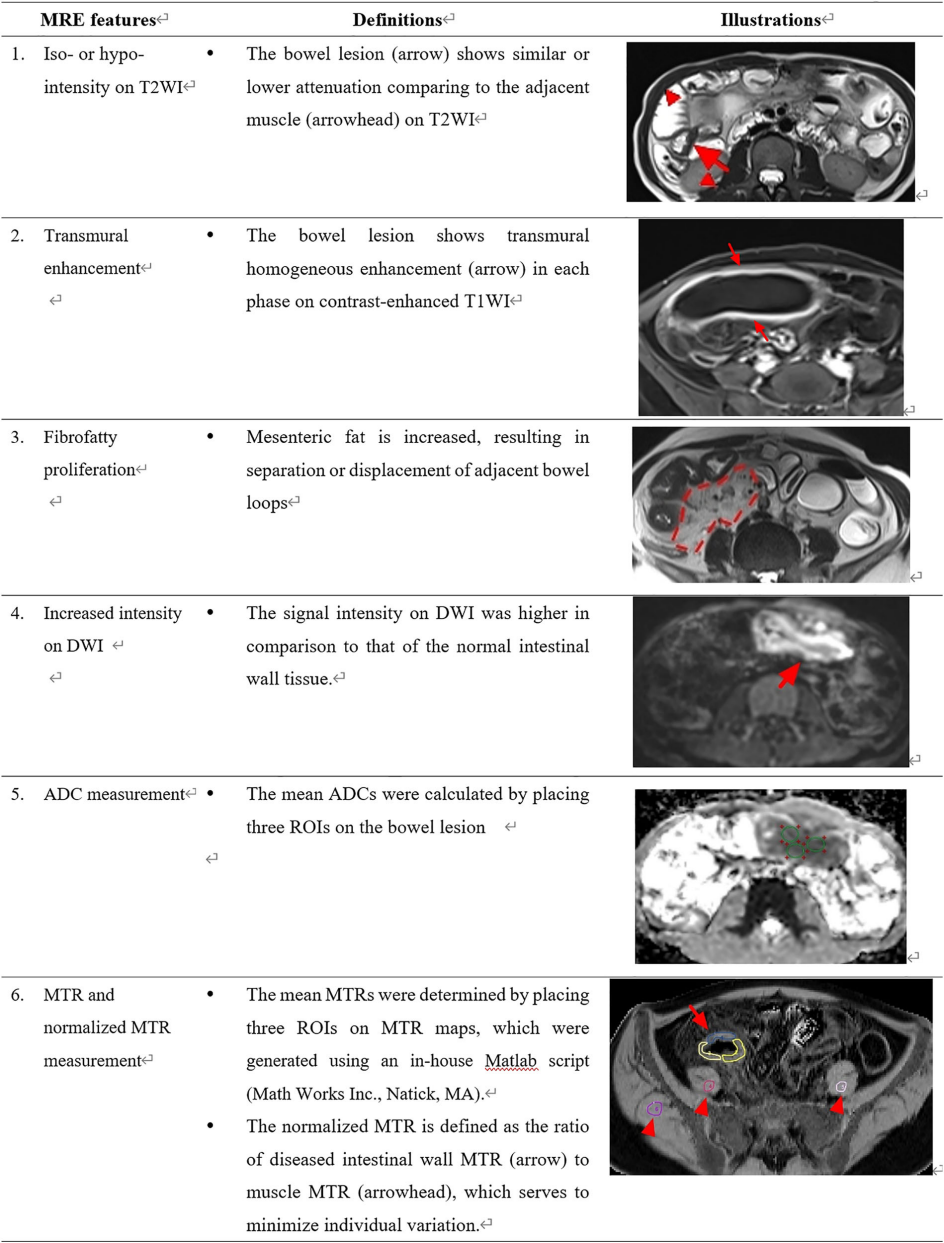
MRE criteria of visual interpretation of intestinal fibrosis in CD. DWI, diffusion-weighted MRI; ADC, apparent diffusion coefficient; MTR, magnetization transfer ratio

The criteria for visual interpretation of intestinal fibrosis, as modified by previous studies [5, 20, 21], were established based on imaging features or parameters derived from T2WI, enhanced T1WI, DWI, ADC, and MTI (Fig. 2). Visual interpretation was independently performed by a gastrointestinal radiologist with 11 years of experience (X.L.), who were blinded to pathological and radiomics data. For criteria 1–4 in Fig. 2, the radiologist documented the interpretation of each imaging feature as either ‘0’ for absence or ‘1’ for presence. As for criteria 5–6 in Fig. 2, the corresponding parameter measurements were recorded. These visual interpretation indexes (Fig. 2) were utilized as features for SVM modelling, and this calculation step was identical to that of the RMs. Similarly, three visual models were constructed by SVM utilizing these visual features from identical sequence combinations (visual model 1: T2WI and enhanced-T1WI; visual model 2: T2WI, enhanced-T1WI, DWI, and ADC); visual model 3: T2WI, enhanced-T1WI, DWI, ADC, and MTI) as the three RMs. We employed ROC analyses to assess their performance. For reproducibility testing, two radiologists with 11 (X.L.) and 3 years’ (Y.W.) of experience, respectively, conducted visual interpretation, and their inter-observer variability was calculated.

### Development and validation of clinical model

Data on clinical features (i.e. age, sex, course of disease, C-reactive protein (CPR), erythrocyte sedimentation rate (ESR), smoking, body mass index, previous surgery history, and Montreal classification for CD) within one week before MRE were retrieved from the electronic medical record system (Table 2). A clinical model was constructed based on the above candidate factors in the training cohort using SVM. The relevance of each clinical feature to the reference standard was assessed. A cross-validation was performed to train the classification model.

**Table 2:** Demographic and clinical characteristics of patients with CD in the training and test cohorts

Characteristics	Training cohort, (*n* = 93)	Test cohort, (*n* = 30)	*p*^a^
Sex, *n* (male/female)	60/33	21/9	0.04
Age, y (mean ± SD)	28.86 ± 8.00	32.32 ± 7.82	0.90
Disease duration, months (median [IQR])	48 (2–240)	42 (1–156)	0.80
Smoking, *n* (%)	13 (13.98)	2 (6.67)	0.58
BMI (median [IQR])	17.92 (14.76–23.78)	17.37 (14.71–24.46)	0.84
Previous surgery history, *n* (%)			0.33
CD-associated bowel resection	9 (9.67)	2 (6.66)	–
Perianal surgery	13 (13.98)	1 (3.33)	–
CRP, mg/L (median [IQR])	21.10 (0.78–101)	34.47 (0.79–153)	0.92
ESR, mm/h (median [IQR])	22.07 (2–104)	23.34 (2–117)	0.55
Montreal classification for CD, *n* (%)			0.80
L1 (ileal)	16 (17.20)	6 (20.00)	–
L2 (colonic)	6 (6.45)	1 (3.33)	–
L3 (ileocolonic)	68 (73.11)	22 (73.33)	–
L4 (isolated upper disease)	17 (18.27)	5 (16.66)	–
B1 (non-stricturing, non-penetrating)	0 (0.00)	0 (0.00)	–
B2 (stricturing)	38 (40.86)	12 (40.00)	–
B3 (penetrating)	55 (59.14)	18 (60.00)	–
*P* (perianal disease modifier)	31 (33.33)	14 (46.66)	–
Type of surgery in the present study, *n* (%)			0.77
Ileocolic resection	21 (22.58)	3 (10.00)	–
Partial small bowel resection	21 (22.58)	7 (23.33)	–
Partial colon resection	20 (21.50)	5 (16.66)	–
Ileocolic + partial small bowel resection	9 (9.67)	5 (16.66)	–
Ileocolic + partial colon resection	1 (1.07)	1 (3.33)	–
Partial small bowel + partial colon resection	19 (20.43)	9 (30.00)	–
Location of specimen, *n*	93	30	0.49
Small bowel, *n* (%)	50 (53.76)	21 (70.00)	–
Colon, *n* (%)	43 (46.24)	9 (30.00)	–
Penetrating diseases, *n*	93	30	0.63
with, *n* (%)	35 (37.63)	14 (46.67)	–
without, *n* (%)	58 (62.37)	16 (53.33)	–

*BMI* body mass index, *CD* Crohn’s disease, *CRP* C-reactive protein, *ESR* erythrocyte sedimentation rate, *IQR* interquartile range, *SD* standard deviation

^a^ Comparison between the training and total test cohorts

### Statistical analysis

The sample size was calculated by the ROC statistical method. The parameters were set based on the following input and assumption: power, 80%; two-sided significance level, 0.05; alternative hypothesis of the AUC, 0.80 compared with the null hypothesis of the AUC, 0.50; and an allocation ratio of sample sizes in none-mild and moderate-severe fibrosis of 1:5 [22]. Therefore, sample sizes of 66 (11 of none-mild, 55 of moderate-severe fibrosis) in the training cohort and 23 (four of none-mild,19 of moderate-severe fibrosis) in the test cohort were sufficient to detect an AUC different from 0.50, with 80% power if the true AUC was > 0.80.

Normally and non-normally distributed data are expressed as the mean ± standard deviation and medians (interquartile ranges), respectively. Either the student’s *t*-test or Welch’s *t*-test was used to calculate the differences among different models and histological scores. Kendall correlation was used to analyze the visual interpretation and histological scores. The DeLong method [23, 24] was used to compare ROC among different models. The clinical utility of the models was measured by decision curve analysis. Statistical analyses were performed using Python and SPSS (version 20; https://www.ibm.com/cn-zh/analytics/spss-statistics-software). A two-sided *p* < 0.05 was considered significant, except for univariate analysis (*p* < 0.10).

## Results

### Demographics

**Fig. 3:**
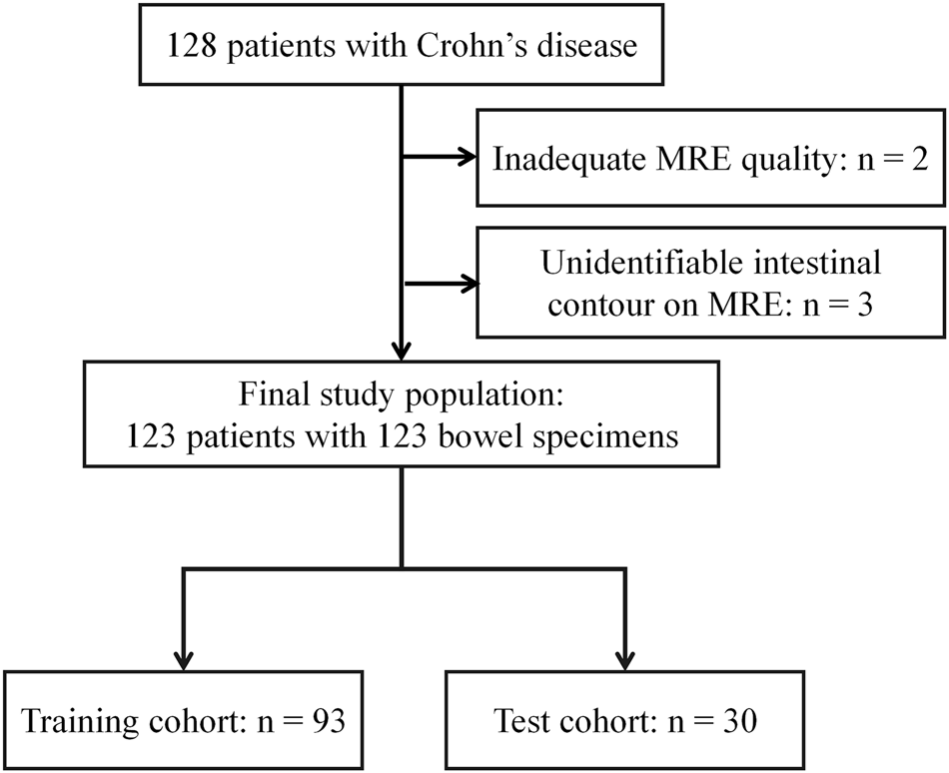
Patient selection diagram

The final included population comprised 123 patients (81 men, 42 women; mean age: 30.26 ± 7.98 years) (Fig. 3). Among them, 93 and 30 patients were selected as the training and test cohorts, respectively. The bowel segments all displayed fibrotic strictures with varying degrees of fibrosis, ranging from mild to severe (Supplementary Table 2). The demographic and clinical characteristics are presented in Table 2.

### RMs performance

#### Radiomic features selection and observer reproducibility

**Fig. 4:**
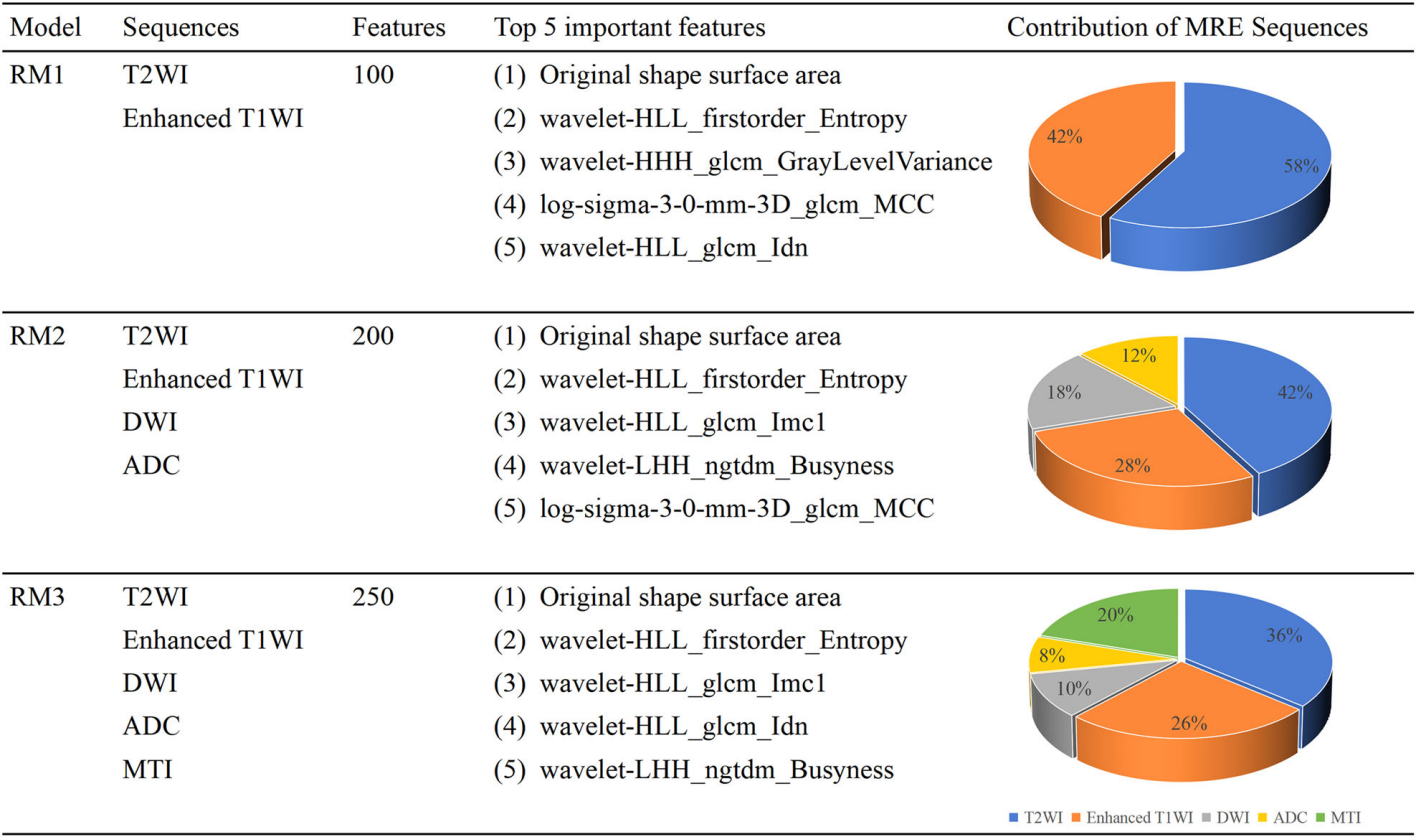
The top five important selected features and the contribution of each MRE sequence in RM1, RM2, and RM3. ADC, apparent diffusion coefficient. DWI, diffusion-weighted imaging; MTI, magnetization transfer MRI; MRE, magnetic resonance enterography; RM, radiomic model

Fifty fibrosis-related features were selected by RFE from each MRE sequence (details in Supplementary materials); these showed robust inter- and intra-observer reproducibility with intraclass correlation coefficients (ICCs) of 0.969–0.998 (all *p* < 0.001) and 0.977–0.985 (all *p* < 0.001), respectively. The top five most important features and the contribution of each MRE sequence in per RM are shown in Fig. 4. The average durations of bowel segmentation, feature extraction, and selection for RM1, RM2, and RM3 were 58 s, 126 s, and 164 s, respectively for each patient.

#### Diagnostic performance of RMs

**Fig. 5:**
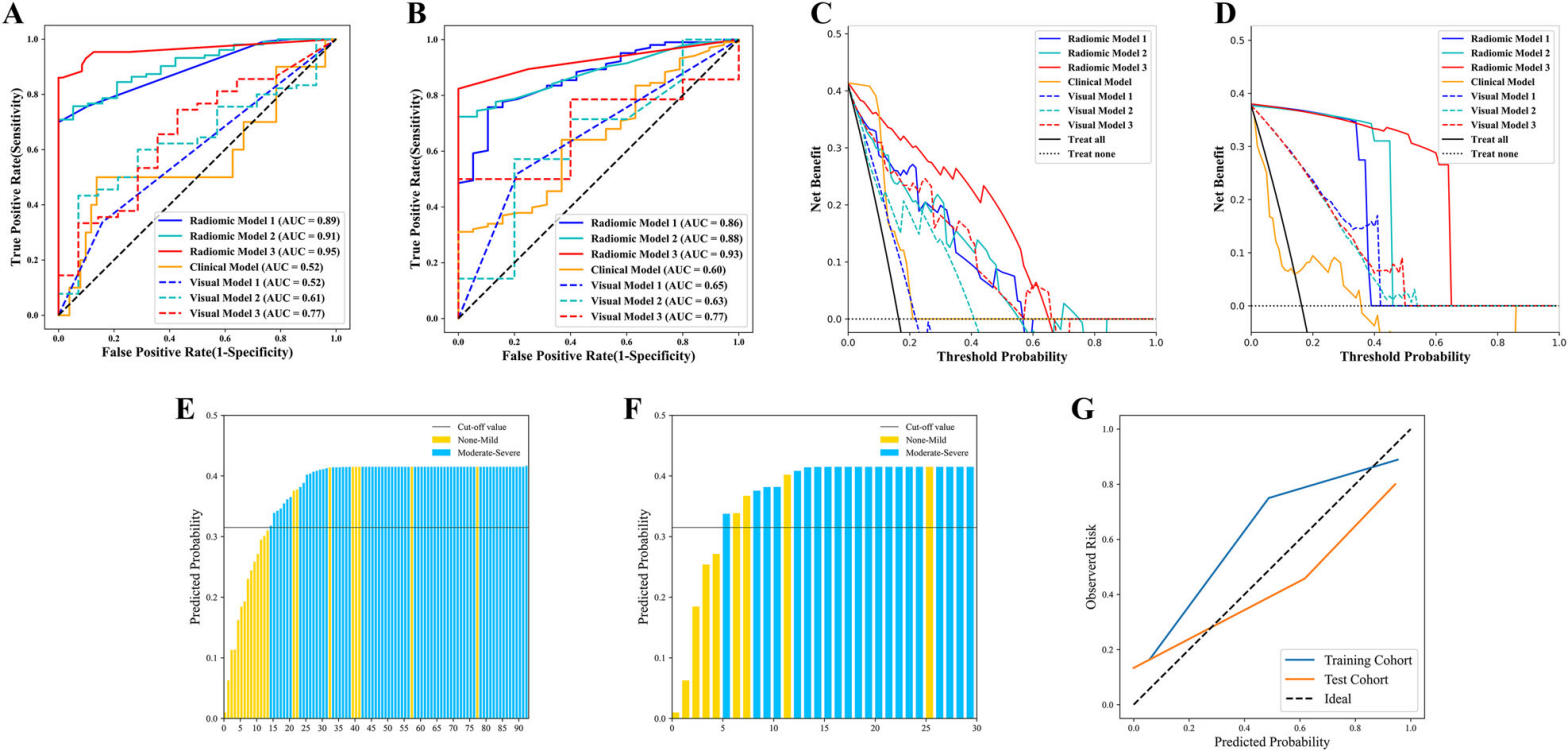
The ROC curves (**A**, **B**), decision curves (**C**, **D**), predicted probabilities (**E**, **F**), and calibration curves (**G**) of the models. The diagnostic performance of the RMs is better than that of both the visual interpretations and clinical model (all *p* < 0.001 according to DeLong’s test) in the training (**A**) and test (**B**) cohorts. RMs have higher net benefits than the two visual interpretation and clinical models in the training (**C**) and test (**D**) cohorts. For the RM3, plots show predicted probabilities with a cut-off value of 0.360 (indicated by the black solid line) in the training (**E**) and test (**F**) cohorts, and the calibration curves (**G**) in both cohorts. RM, radiomic model; ROC, receiver operating characteristic; AUC, area under the receiver operating characteristic curve

In the training cohort, RM1, RM2, and RM3 had AUCs of 0.89 (*p* = 0.001), 0.91 (*p* = 0.01), and 0.95 (*p* = 0.009), respectively, demonstrating a gradual increase in diagnostic accuracy (Fig. 5A). In the test cohort, RM1, RM2, and RM3 had AUCs of 0.86 (*p* = 0.001), 0.88 (*p* = 0.001), and 0.93 (*p* = 0.02), respectively, showing a similar increasing trend in AUC as observed in the training cohort (Fig. 5B and Table 3). The NRI and IDI for RM2 compared to RM1 (*p* = 0.045, DeLong’s test) were 0.140 (95% CI: 0.084–0.203) and 0.072 (95% CI: 0.045–0.097), respectively. Comparing RM3 with RM2 (*p* = 0.01, DeLong’s test), the NRI and IDI were 0.100 (95% CI: 0.066–0.170) and 0.100 (95% CI: 0.061–0.135), respectively. Decision curve analysis further confirmed that the addition of more specific sequences gradually improved the diagnostic performance of the three RMs, providing better net benefits for predicting intestinal fibrosis (Fig. 5C, D).

**Table 3:** Diagnostic performance of the RMs, radiologist’s visual models and clinical models for detection of moderate to severe fibrosis in the training and test cohorts

Model	Accuracy (%)	Sensitivity (%)	Specificity (%)	PPV (%)	NPV (%)	AUC (95% CI)	*p*
Training cohort, (*n* = 93)^a^							
RM1	82 (76/93)	78 (62/79)	100 (14/14)	100 (62/62)	45 (14/31)	0.89 (0.74–0.93)	0.001
Visual model 1	38 (35/93)	30 (24/79)	79 (11/14)	89 (24/27)	17 (11/66)	0.52 (0.38–0.66)	0.26
RM2	85 (79/93)	82 (65/79)	100 (14/14)	100 (65/65)	50 (14/28)	0.91 (0.79–0.97)	0.01
Visual model 2	54 (50/93)	47 (37/79)	93 (13/14)	97 (37/38)	24 (13/55)	0.61 (0.54–0.73)	0.02
RM3	91 (85/93)	90 (71/79)	100 (14/14)	100 (71/71)	64 (14/22)	0.95 (0.86–0.99)	0.009
Visual model 3	76 (71/93)	77 (61/79)	71 (10/14)	94 (61/65)	36 (10/28)	0.76 (0.66–0.87)	0.001
Clinical model	41 (38/93)	33 (26/79)	86 (12/14)	93 (26/28)	19 (12/65)	0.52 (0.44–0.70)	0.12
Test cohort, (*n* = 30)^a^							
RM1	77 (23/30)	76 (19/25)	80 (4/5)	95 (19/20)	40 (4/10)	0.86 (0.79–0.94)	0.001
Visual model 1	60 (18/30)	56 (14/25)	80 (4/5)	93 (14/15)	27 (4/15)	0.65 (0.53–0.78)	0.56
RM2	80 (24/30)	76 (19/25)	100 (5/5)	100 (19/19)	46 (5/11)	0.88 (0.82–0.95)	0.001
Visual model 2	53 (16/30)	52 (13/25)	60 (3/5)	87 (13/15)	20 (3/15)	0.63 (0.48–0.77)	0.042
RM3	87 (26/30)	84 (21/25)	100 (5/5)	100 (21/21)	56 (5/9)	0.93 (0.87–0.98)	0.02
Visual model 3	83 (25/30)	84 (21/25)	80 (4/5)	95 (21/22)	50 (4/8)	0.77 (0.65–0.89)	0.002
Clinical model	57 (17/30)	52 (13/25)	80 (4/5)	93 (13/14)	25 (4/16)	0.60 (0.47–0.72)	0.13

Data in parentheses are numerators and denominators

RMs and visual models were based on different MR sequence combinations: RM1 or visual model 1 (T2WI and contrast-enhanced T1WI), RM2 or visual model 2 (T2WI, contrast-enhanced T1WI, and diffusion-weighted imaging [DWI], apparent diffusion coefficient [ADC]), RM3 or visual model 3 (T2WI, contrast-enhanced T1WI, DWI, ADC, and magnetization transfer MRI)

*AUC* area under the receiver operating characteristic curve, *CI* confidence interval, *NPV* negative predictive value, *PPV* positive predictive value, *RM* radiomic model

^a^ Number of resected bowel segments

RM3 had the highest diagnostic efficacy, and its predicted probability for each intestinal lesion is shown in Fig. 5E, F. Moreover, the Hosmer–Lemeshow test indicated that RM3 was effective and robust with a $x$^2^ of 8.541 (*p* = 0.32) and 12.140 (*p* = 0.58) in the training and test cohorts, respectively (Fig. 5G).

#### The diagnostic performance of RMs within different subgroups

In both training and test cohorts, there were no significant differences in the performance of RMs for diagnosing fibrosis between different inflammatory bowel segments, strictures in small or colonic, different MRI scanners (Magnetom Trio or Prisma; Siemens Healthineers), and bowel segments with or without penetrating diseases (all *p* > 0.05) (Supplementary Tables 4–7), indicating the high reliability and stability of these three RMs for diagnosing bowel fibrosis.

### Visual interpretation performance

The performance of visual models 1 and 2 in distinguishing degrees of intestinal fibrosis was found to be poor, both in the training (AUC = 0.52, 0.61, respectively) and test cohort (AUC = 0.65, 0.63, respectively) (all *p* > 0.05). The inclusion of MTI measurements improved the diagnostic performance of visual model 3 both in the training (AUC = 0.77, *p* < 0.001) and test cohorts (AUC = 0.77, *p* = 0.002) (Fig. 5A, B).

The inter-observer agreement assessment for MRE features showed poor results for ‘T2WI iso-/hypo-intensity’, ‘transmural enhancement’, and ‘fibrofatty proliferation’ with ICCs of 0.331 (*p* < 0.001), 0.114 (*p* = 0.10), and 0.275 (*p* < 0.001), respectively, while the assessment for other features including ‘increased intensity on DWI’, ADC, MT-ratio, and normalized MT-ratio had good agreement with ICCs of 0.772, 0.768, 0.855, and 0.824, respectively (all *p* < 0 .001).

### Clinical model performance

The clinical model was constructed using SVM based on univariate analysis, selecting CPR and ESR from the nine clinical features. However, this clinical model failed to differentiate between the different degrees of intestinal fibrosis in both the training (AUC = 0.52, *p* = 0.12) and test (AUC = 0.60, *p* = 0.13) cohorts.

### Comparing the diagnostic performance of the RMs with those of the clinical model and visual’ interpretation

In the training and test cohorts, the RMs all performed significantly better than the corresponding visual interpretation and the clinical model in evaluating intestinal fibrosis (DeLong’s test, all *p* < 0.05) (Fig. 5A, B).

Decision curve analysis showed that the RMs provided a better net benefit for predicting intestinal fibrosis than the clinical model and visual interpretation in the training (Fig. 5C) and test (Fig. 5D) cohorts.

## Discussion

To address the lack of non-invasive and non-irradiating tools for precisely detecting transmural fibrosis, we developed three multi-sequence MRE-based RMs, which showed satisfactory efficacy in distinguishing moderate-severe from none-mild intestinal fibrosis in CD. RM1, constructed using basic MRI sequences, already had moderate diagnostic accuracy. With more specific MRI sequences (DWI or MTI) added, the diagnostic performance of RM1, RM2, and RM3 improved gradually from moderate to excellent. Moreover, these three RMs ran steadily in an independent test cohort and in different subgroups, indicating their high reliability and stability. Additionally, all RMs outperformed the MR-visual interpretation and the clinical model in diagnosing bowel fibrosis.

MRE, with a multi-sequence combination, could reflect various histopathological features of CD intestinal fibrosis. The combination of MTI and T2WI has been reported as a viable method for assessing bowel fibrosis [9]. However, conventional image analysis of MRE typically evaluates bowel features in only one image slice or using a limited ROI, assuming the homogeneity of bowel features over the entire image. This may explain why traditional imaging interpretation remains insufficient for detecting intestinal fibrosis accurately or produces inconsistent results in its evaluation [6–9]. The application of radiomic analysis in MRE offers a novel perspective for the characterization of intestinal fibrosis in CD, providing more objective and reproducible transmural measurements.

We proposed three RMs with different combinations of sequences, taking into account the technical feasibility of their implementation and the acceptable scanning time for patients with CD. RM1 with only basic MR sequences had already achieved moderate diagnostic efficacy, demonstrating the potential of radiomics in digging fibrosis-related information hidden in MRE images. This finding is consistent with previous reports on texture analysis using conventional sequences [10, 11]. Therefore, RM1 is a great option for basic healthcare hospitals that do not perform DWI and MTI scans due to the extended scan time or unavailability of the sequences. Encouragingly, after adding DWI and ADC to RM1 to construct RM2, the diagnostic accuracy improved considerably. This positive result could be because DWI with ADC can reflect the change in extracellular water molecule diffusion that is affected by collagen deposition within the fibrotic bowel segment [25, 26]. As expected, RM3, which combines the MTI, diffusion, and conventional sequences, had the highest diagnostic efficiency among the three RMs. A previous animal study reported both T2WI texture analysis and MTI can detect established bowel fibrosis [11]. MTI enables the indirect quantification of macromolecule concentrations in tissues, reflecting the severity of collagen deposition within the bowel wall regardless of coexisting inflammation [7, 9]. Applying radiomics to the analysis of MTI images further increases its potential for characterizing intestinal fibrosis. Thus, RM3 exhibited the best diagnostic performance.

Our RMs were developed using radiomic features with good interobserver consistency and robust classification performance in both the training and test cohorts. Furthermore, their efficacy was stable and unaffected by factors such as concomitant inflammation severity. Since inflammation and fibrosis often co-exist within the same intestinal segment, it is important that RMs assess the degree of fibrosis independent of inflammation. Moreover, RMs are unaffected by differences in intestinal location, and the presence of penetrating lesions. No significant differences in the performance of the RMs were observed when utilizing two distinct MRI scanners.

The three RMs demonstrated superior efficacy in grading intestinal fibrosis compared to the visual models using traditional imaging interpretation of corresponding MR sequences. This suggests that RMs can enhance the ability of radiologists to assess fibrosis. This result was consistent with our previous CTE-based radiomic study [15], as it was easier for computer algorithms to accurately identify the details of intestinal fibrosis. On the other hand, while visual models based on conventional and/or diffusion sequences showed poor performance in evaluating fibrosis, incorporating MTI improved their efficacy. This may be attributed to the superior ability of MTI in assessing fibrosis compared to conventional sequences and DWI, which aligned with previous research findings [7, 9]. Additionally, the poor agreement between observers’ interpretation of T2WI and enhanced-T1WI features may be ascribed to variations in their levels of expertise. Similarly, the RMs were superior to the clinical model in diagnosing fibrosis. The fact that this clinical model targeted the whole patient rather than the specific inflamed intestine is the major reason for its inefficiency.

Our study has several limitations. First, the requirement for resected specimens may have resulted in selection bias toward enroling more patients with advanced CD and relatively fewer patients with none-mild fibrosis for analysis. Second, precise point-by-point correlations between MRE and surgical specimens were difficult to obtain due to bowel peristalsis. With a relatively short interval between MRE and surgery along with pre-scanning hypotonic bowel preparation, we achieved region-by-region correlations between MRE and specimens based on the identification of anatomical structure or gross lesion, as described in a prior study [7]. Lastly, our study had a relatively limited sample size without including multicenter data. The need for a standardized MRE examination, especially with qualified MTI, hindered the inclusion of eligible centers and samples. However, to our best knowledge, this study included the largest sample size reported to date in the field of MRE-based radiomics analysis of bowel fibrosis in CD. These promising results from our center suggest the necessity and feasibility of future prospective multicenter studies.

In conclusion, multi-sequence MRE-based RMs are a valuable tool for accurately characterizing intestinal fibrosis in CD and can significantly enhance radiologists’ diagnostic ability. Our three RMs, constructed using different combinations of conventional, DWI and MTI sequences, meet diverse technical feasibility requirements for implementation in various institutions for patients with CD.

### Supplementary information

ELECTRONIC SUPPLEMENTARY MATERIAL

## Data Availability

The data that support the findings of this study are available from the corresponding authors with a signed data access agreement. The raw image data are not publicly available because they contain sensitive information that could compromise patient privacy. We are pleased to share all the codes for feature extraction and model construction upon request for research purpose.

## Abbreviations

ADC: Apparent diffusion coefficient
AUC: Area under the receiver operating characteristic curve
CD: Crohn’s disease
DWI: Diffusion-weighted imaging
ICC: Intraclass correlation coefficient
IDI: Integrated discrimination improvement
MRE: Magnetic resonance enterography
MTI: Magnetization transfer magnetic resonance imaging
NRI: Net reclassification improvement
RFE: Recursive feature elimination
RM: Radiomic model
SVM: Support vector machines
