# Supplementary material for: MRI radiomics enhances radiologists’ ability for characterizing intestinal fibrosis in patients with Crohn’s disease
Source: Insights Imaging. 2024 Jun 28;15:165. doi: 10.1186/s13244-024-01740-6 (PMC11213838; doi:10.1186/s13244-024-01740-6)
Supplement: Supplementary file 1 — ELECTRONIC SUPPLEMENTARY MATERIAL [file 13244_2024_1740_MOESM1_ESM.pdf]

MRI Radiomics Enhances Radiologists' Ability for Characterizing Intestinal Fibrosis in Patients with Crohn's Disease

ELECTRONIC SUPPLEMENTARY MATERIAL

Region-by-region location between MR enterography and surgical specimens and histopathological sections

Region-by-region matching radiological and surgical analyses of each surgical specimen were performed by a radiologist (C.S.) with more than 20 years of experience in abdominal MR enterography (MRE) and a surgeon (Z.C.) with 11 years of experience in intestinal resection for Crohn’s disease (CD) in the operating room. A bowel segment was obtained from each patient. For matched analysis between MRE and surgical specimens, each lesion was located according to its features (e.g., location, length, stricture, and fistula) or adjacent anatomic landmarks (e.g., ileocecal valve, appendix) [1-2]. The resected bowel lesions were examined histopathologically by a specialist gastrointestinal pathologist (Z.Y.) with 12 years of experience (Supplementary Tables 1-2).

MR enterography protocol

The bowel was prepared as described previously[2]. Briefly, a polyethylene glycol electrolyte solution was ingested 6–8 h before MRE, followed by an oral contrast agent (1600–2000 mL of 2.5% mannitol solution) 1 h before MRE. Raceanisodamine hydrochloride (20 mg, Minsheng Pharmaceutical Group Co.) was injected intramuscularly into the buttocks 10 min before MRE.

MRE was performed on two 3-T MR system (Magnetom Trio or Prisma; Siemens Healthineers) equipped with 12- (Trio) or 18-channel phased-array body coils (Prisma), including (1) T2-weighted imaging; (2) diffusion-weighted magnetic resonance imaging (DWI, b values of 50, 400, 800 s/mm^2; the apparent diffusion coefficient map was reconstructed from the original DWI data); (3) Magnetisation transfer magnetic resonance imaging (MTI) and (4) T1-weighted imaging before and at 30s, 60s, 90s, 2 min, 3.5 min, and 7 min after an intravenous injection of 0.2 mL/kg gadopentetate dimeglumine (Gd-DTPA) (Beilu Pharmaceuticals, Beijing, China) at a rate of 2 mL/s (Trio) or 0.1 mL/kg gadobutrol (Bayer AG, Leverkusen, Germany) at a rate of 1.5 mL/s (Prisma).

Development and validation of MRE-based RMs

Features selection and RM development

To avoid overfitting and to select the best parameters for the diagnostic model, we conducted 10-fold cross-validation in the training cohort. In the training procedure of this 10-fold cross-validation, a grid search method was used to determine the optimal parameters made of kernel type (type of kernel function), C (inverse of regularisation strength), gamma (bandwidth of kernel function, radius of hypercircle), and max_iter (maximum number of iterations required for the solvers to converge) in the SVM classifier. The overall prediction performance in the training cohort was assessed based on all probabilities of the testing samples in each fold during cross-validation. Model development was performed in Python (version 3.6; https://www.python.org/) using the Scikit-learn package (version 0.22; https://www.scikit-learn.org/).

RMs performance

Radiomic features selection & observer reproducibility

A total of 1130 radiomic features were extracted from each MRE sequence, and 400 features with a variance close to zero were eliminated. For the remaining 730 features, the median intraclass correlation coefficients (ICCs) for inter- and intra-observer agreement assessments were 0.972 (quartile range, 0.957–0.998) and 0.966 (quartile range, 0.961–0.985) respectively, justifying the reliability of the radiomic features.

Supplementary Table 1. Histologic scores for intestinal fibrosis and inflammation

Score	Fibrosis	Inflammation
None-mild		
0	No fibrosis	No inflammation or distortion
1	Minimal fibrosis in submucosa or subserosa	Lamina propria inflammation only
2	Increased submucosal fibrosis, septa into muscularis propria	Submucosal foci of inflammation
Moderate-severe		
3	Septa through muscularis propria, increase in subserosal collagen	Foci of transmural inflammation
4	Significant transmural scar, marked subserosal collagen	Significant, dissecting, confluent transmural inflammation

Supplementary Table 2. Histological fibrosis and inflammation scores in the training and test cohorts

	Training cohort	Test cohort	*P*†
Bowel segments, n	93	30	--
Fibrosis score, median [IQR]	3 (2–3)	3 (2–4)	<0.001
1	2	1	--
2	12	4	--
3	44	16	--
4	35	9	--
Inflammation score, median [IQR]	3 (3–4)	3 (2–3)	<0.001
1	1	0	--
2	8	8	--
3	31	9	--
4	53	13	--

IQR, interquartile range † Comparison between the training and test cohorts.

Supplementary Table 3. Visual interpretation of intestinal fibrosis on MRE in the training and test cohorts

MRE features	Training cohort (n=93)	Test cohort (n=30)	*P*†
T2WI iso- to hypo-intensity compared to adjacent muscle			<0.001
0	60	18	
1	33	12	
Transmural enhancement			<0.001
0	63	22	
1	30	8	
Widening of Mesenteric fat			<0.001
0	40	14	
1	53	16	
DWI			<0.001
0	5	4	
1	88	26	
ADC (mean ± SD, 10^{-3} mm^2/s)	1216 ± 327	1001 ± 572	<0.001
MTR (mean ± SD)	34 ± 15	36 ± 13	<0.001
Normalized MTR (mean ± SD)	0.69 ± 0.30	0.71 ± 0.26	<0.001

Note. —The observation result of each MRE feature was recorded as a score, of which ‘0’ refers to absence and ‘1’ to presence. MRE, magnetic resonance imaging. DWI,

diffusion-weighted MRI; ADC, apparent diffusion coefficients; MTR, magnetization transfer ratio † Comparison between the training and total test cohort

Supplementary Table 4. Diagnostic performances of the radiomic models dependent on inflammation severity in the training and test cohorts

Model	Accuracy (%)	Sensitivity (%)	Specificity (%)	AUC (95% CI)	DeLong test‡ (*P*)
Training cohort (n= 93) †					
Inflammatory severity					
None–mild (n= 9)					
RM1	78 (7/9)	60 (3/5)	100 (4/4)	0.80 (0.75–0.86)	0.56
RM2	89 (8/9)	80 (4/5)	100 (4/4)	0.90 (0.86–0.95)	0.42
RM3	100 (9/9)	100 (5/5)	100 (4/4)	1.00 (1.00–1.00)	0.67
Moderate–severe (n= 84)					
RM1	81(68/84)	78 (58/74)	100 (10/10)	0.86 (0.80–0.91)	--
RM2	82(69/84)	80 (59/74)	100 (10/10)	0.86 (0.81–0.91)	--
RM3	88(74/84)	87 (64/74)	100 (10/10)	0.93 (0.88–0.98)	--
Test cohort (n=30) †					
None–mild (n= 8)					
RM1	88(7/8)	83 (5/6)	100 (2/2)	0.92 (0.89–0.95)	0.84
RM2	75(6/8)	67 (4/6)	100 (2/2)	0.83 (0.80–0.91)	0.64
RM3	100(8/8)	100 (6/6)	100 (2/2)	1.00 (1.00–1.00)	0.55
Moderate–severe (n= 22)					

RM1	73(16/22)	68 (13/19)	3/3(1.000)	0.84 (0.79–0.90)	--
RM2	77(17/22)	74 (14/19)	3/3(1.000)	0.87 (0.81–0.95)	--
RM3	82(18/22)	79(15/19)	3/3(1.000)	0.90 (0.83–0.98)	--

Note. —Data in parentheses are numerators and denominators. RMs were based on different MR sequence combinations: RM1 (T2WI and enhanced-T1WI), RM2 (T2WI, enhanced-T1WI, and diffusion-weighted imaging [DWI], apparent diffusion coefficient [ADC]), and RM3 (T2WI, enhanced-T1WI, DWI, ADC, and magnetisation transfer MRI)

RM, Radiomic Model; CI, confidence interval; AUC, area under the receiver operating characteristic curve

†Number of resected bowel segments. ‡Comparison of AUC for diagnosing fibrosis between the none-mild and moderate-to-severe inflammatory bowel segments.

Supplementary Table 5. Diagnostic performance of the radiomic models dependent on location of Crohn's disease in the training and test cohorts

Model	Accuracy (%)	Sensitivity (%)	Specificity (%)	AUC (95% CI)	DeLong test‡ (P)
Training cohort (n= 93) †					
Lesion locations					
Small bowel strictures (n= 50)					
RM1	82 (41/50)	80 (36/45)	100 (5/5)	0.86 (0.76–0.94)	0.32
RM2	80 (40/50)	78 (35/45)	100 (5/5)	0.82 (0.71–0.93)	0.62
RM3	88 (44/50)	87 (39/45)	100 (5/5)	0.90 (0.81–0.98)	0.74
Colonic strictures (n= 43)					
RM1	91 (39/43)	88 (30/34)	100 (9/9)	0.95 (0.85–1.00)	--
RM2	88 (37/43)	82 (28/34)	100 (9/9)	0.93 (0.80–0.98)	--
RM3	88 (38/43)	83 (29/34)	100 (9/9)	0.90 (0.82–0.96)	--
Test cohort (n=30) †					
Small bowel strictures (n= 21)					
RM1	71 (15/21)	65 (11/17)	100 (4/4)	0.82 (0.66–0.89)	0.75
RM2	76 (16/21)	71 (12/17)	100 (4/4)	0.85 (0.78–0.93)	0.54
RM3	81 (17/21)	76 (13/17)	100 (4/4)	0.88 (0.87–0.91)	0.63
Colonic strictures (n= 9)					

RM1	67 (6/9)	63 (5/8)	100 (1/1)	0.81 (0.78–0.86)	--
RM2	78 (7/9)	75 (6/8)	100 (1/1)	0.88 (0.81–0.95)	--
RM3	89 (8/9)	88 (7/8)	100 (1/1)	0.94 (0.88–0.98)	--

Note. —Data in parentheses are numerators and denominators. RMs were based on different MR sequence combinations: RM1 (T2WI and enhanced-T1WI), RM2 (T2WI, enhanced-T1WI, and diffusion-weighted imaging [DWI], apparent diffusion coefficient [ADC]), and RM3 (T2WI, enhanced-T1WI, DWI, ADC, and magnetisation transfer MRI)

RM, Radiomic Model; CI, confidence interval; AUC, area under the receiver operating characteristic curve

†Number of resected bowel segments. ‡Comparison of AUC for diagnosing fibrosis between the small bowel and colonic strictures.

Supplementary Table 6. Diagnostic performance of the radiomic models in different magnetic resonance scanners in the training and test cohorts

Model	Accuracy (%)	Sensitivity (%)	Specificity (%)	AUC (95% CI)	DeLong test‡ (*P*)
Training cohort (n= 93) †					
Magnetic resonance scanners					
MR1 (n= 37)					
RM1	78 (29/37)	70 (19/27)	100 (10/10)	0.85 (0.81–0.90)	0.65
RM2	87 (32/37)	81 (22/27)	100 (10/10)	0.93 (0.87–0.97)	0.21
RM3	76 (28/37)	67 (18/27)	100 (10/10)	0.84 (0.79–0.91)	0.56
MR2 (n= 56)					
RM1	77 (43/56)	75 (39/52)	100 (4/4)	0.87 (0.82–0.93)	--
RM2	75 (42/56)	73 (38/52)	100 (4/4)	0.85 (0.80–0.90)	--
RM3	95 (53/56)	94 (49/52)	100 (4/4)	0.97 (0.88–1.00)	--
Test cohort (n=30) †					
MR1 (n= 25)					
RM1	76 (19/25)	75 (18/24)	100 (1/1)	0.88 (0.83–0.87)	0.81
RM2	80 (20/25)	79 (19/24)	100 (1/1)	0.90 (0.77–0.91)	0.10
RM3	72 (18/25)	71 (17/24)	100 (1/1)	0.85 (0.79–0.90)	0.22
MR2 (n= 5)					

RM1	60 (3/5)	100 (1/1)	50 (2/4)	0.75 (0.68–0.78)	--
RM2	80 (4/5)	100 (1/1)	75 (3/4)	0.88 (0.86–0.89)	--
RM3	100 (5/5)	100 (1/1)	100 (4/4)	1.00 (1.00–1.00)	--

Note. —Data in parentheses are numerators and denominators. RMs were based on different MR sequence combinations: RM1 (T2WI and enhanced-T1WI), RM2 (T2WI, enhanced-T1WI, and diffusion-weighted imaging [DWI], apparent diffusion coefficient [ADC]), and RM3 (T2WI, enhanced-T1WI, DWI, ADC, and magnetisation transfer MRI)

MR1, Magnetom Trio; MR2, Magnetom Prisma; RM, Radiomic Model; CI, confidence interval; AUC, area under the receiver operating characteristic curve

†Number of resected bowel segments. ‡Comparison of AUC for diagnosing fibrosis between MR1 and MR2.

Supplementary Table 7. Diagnostic performance of the radiomic models in bowel strictures with and without penetrating diseases in the training and test cohorts

Model	Accuracy (%)	Sensitivity (%)	Specificity (%)	AUC (95% CI)	DeLong test‡ (P)
Training cohort (n= 93) †					
Penetrating diseases					
Without penetrating diseases (n= 58)					
RM1	78 (45/58)	74 (37/50)	100 (8/8)	0.85 (0.81–0.91)	0.24
RM2	74 (43/58)	70 (35/50)	100 (8/8)	0.81 (0.77–0.86)	0.59
RM3	88 (51/58)	86 (43/50)	100 (8/8)	0.93 (0.87–0.97)	0.36
With penetrating diseases (n= 35)					
RM1	86 (30/35)	83 (24/29)	100 (6/6)	0.91 (0.87–0.97)	--
RM2	83 (29/35)	79 (23/29)	100 (6/6)	0.90 (0.87–0.96)	--
RM3	80 (28/35)	76 (22/29)	100 (6/6)	0.88 (0.82–0.93)	--
Test cohort (n=30) †					
Without penetrating diseases (n= 16)					
RM1	69 (11/16)	58 (7/12)	100 (4/4)	0.79 (0.72–0.89)	0.43
RM2	75 (12/16)	67 (8/12)	100 (4/4)	0.83 (0.79–0.90)	0.15
RM3	88 (14/16)	83 (10/12)	100 (4/4)	0.92 (0.88–0.98)	0.70
With penetrating diseases (n= 14)					

RM1	86 (12/14)	85 (11/13)	100 (1/1)	0.92 (0.88–0.98)	--
RM2	79 (11/14)	77 (10/13)	100 (1/1)	0.89 (0.85–0.96)	--
RM3	71 (10/14)	69 (9/13)	100 (1/1)	0.85 (0.83–0.93)	--

Note. —Data in parentheses are numerators and denominators. RMs were based on different MR sequence combinations: RM1 (T2WI and enhanced-T1WI), RM2 (T2WI, enhanced-T1WI, and diffusion-weighted imaging [DWI], apparent diffusion coefficient [ADC]), and RM3 (T2WI, enhanced-T1WI, DWI, ADC, and magnetisation transfer MRI)

RM, Radiomic Model; CI, confidence interval; AUC, area under the receiver operating characteristic curve

†Number of resected bowel segments. ‡Comparison of AUC for diagnosing fibrosis between patients with and without penetrating disease.

References

1. Wagner M, Ko HM, Chatterji M, et al. Magnetic resonance imaging predicts histopathological composition of ileal Crohn's Disease. J Crohns Colitis 2018;12:718-729.

2. Li XH, Mao R, Huang SY et al. Characterization of Degree of Intestinal Fibrosis in Patients with Crohn Disease by Using Magnetization Transfer MR Imaging. Radiology 2018;287(2):494-503.
